# Supplementary material for: Relating Molecular Dynamics Simulations to Functional Activity for Gly-Rich Membranolytic Helical Kiadin Peptides
Source: Pharmaceutics. 2023 May 8;15(5):1433. doi: 10.3390/pharmaceutics15051433 (PMC10220883; doi:10.3390/pharmaceutics15051433)
Supplement: Supplementary file 1 [file pharmaceutics-15-01433-s001.zip › pharmaceutics-2336337-supplementary.pdf]

Relating Molecular Dynamics Simulations to Functional Activity for Gly-Rich Membranolytic Helical Kiadin Peptides

Tomislav Rončević [1,#], Matko Maleš [2,#], Yogesh Sonavane [3], Filomena Guida [4], Sabrina Pacor [4], Alessandro Tossi [4] and Larisa Zoranić [3,*]

[1] University of Split, Faculty of Science, Department of Biology, 21000 Split, Croatia
[2] University of Split, Faculty of Maritime Studies, 21000 Split, Croatia
[3] University of Split, Faculty of Science, Department of Physics, 21000 Split, Croatia
[4] University of Trieste, Department of Life Sciences, 34127 Trieste, Italy
* Correspondence: larisa.zoranic@pmfst.hr or larisaz@pmfst.hr ; Tel.: 00385-21-619-221

[#] Equal contribution

Supplementary data

Details of the MD equilibration process and analysis

Figure S1A. Analytical RP-HPLC of kiadin peptides.

Figure S1B: ES-MS spectra for kiadins peptides.

Figure S2. Snapshots of the initial conditions for MD simulations of kiadin peptides.

Figure S3. Helical wheel projections of kiadin peptides.

Figure S4. Visual representation of the 3D-HM results for kiadins at 0 ns simulation time.

Figure S5A. Representation of the 3D-HM results at 300 ns simulation time for DLPC membranes.

Figure S5B. Representation of the 3D-HM results at 300 ns simulation time for POPE:POPG membranes.

Figure S6A. Number of contacts as a function of simulation time between all peptide atoms and phospholipid phosphate atoms for DLPC systems.

Figure S6B. Number of contacts as a function of simulation time between all peptide atoms and phospholipid phosphate atoms for POPE:POPG systems.

Figure S7. Average number of contacts between all atoms belonging to the peptide and phosphate atoms of phospholipids averaged over the last 100 ns of simulation time.

Figure S8. Average number of contacts between individual peptide Lys side-chains and membrane phosphate atoms averaged over the last 100 ns of simulation time.

Figure S9A. The time evolution of the secondary structuring for 300 ns in DLPC systems.

Figure S9B. The time evolution of the secondary structuring for 300 ns in POPE:POPG systems.

Figure S10. Percent α-helicity for each kiadin averaged over 300 ns simulation time.

Table S1. 3D-HM calculations.

Figure S11. Strength of 3D-HM and angle between 3D-HM vector and +z axis (axis perpendicular to the membrane surface), calculated at 0 ns and 300 ns simulation time.

Figure S12A. Density profiles for kiadin peptides with DLPC membrane sampled over the last 100 ns simulation time.

Figure S12B. Density profiles for kiadin peptides with POPE:POPG membrane sampled over the last 100 ns simulation time.

Table S2. Qualitative comparison of MD simulation, biophysical data and biological activities.

Details of the MD equilibration process

Both types of membranes were subjected to molecular dynamics simulations (DLPC for 100 ns; POPE:POPG for 250 ns) under the same conditions and parameters as subsequently used for the simulations in the presence of peptides. These resulted in an average area per lipid of 63.6 $Å^2$ for the DLPC membrane and 60.4 $Å^2$ for the POPE:POPG membrane, which is close to reported experimental values of 63.2 ± 0.5 $Å^2$ for DLPC at 300 K, and 61.5 ± 0.2 $Å^2$ for POPE:POPG at 310 K in the literature.

A temperature annealing procedure was used to equilibrate the peptide-membrane system to the reference temperature and pressure (T = 310 K; p = 1 ATM), ensuring that lipids are in the fluid phase (T_M = 272 K for DLPC, 300 K for POPE and 269 K for POPG). During equilibration, a position restraint algorithm was used to fix peptide heavy atoms and preserve their α-helical structure.

Details of the MD analysis

Post-run analyses were performed mainly by using Gromacs utilities, such as the *gmx* routine *mindist*, which computes the number of contacts and minimum distance between one group and a number of other groups [1]. Here we calculated the number of contacts occurring within a 0.6 nm cut-off value using these criteria: *i*) if one or more atoms in the peptide comes within the cut-off distance to a phospholipid P atom in the nearest leaflet this is counted as one contact; *ii*) if any atom in the side-chain of each Lys residue comes within the contact distance of a phosphorous atom of phospholipids in the nearest leaflet, this is counted as one, taking into account the possibility of multiple contacts.

Density profiles were calculated for the last 100 ns of simulation time, using the *gmx density* utility: *i)* the distribution of P atoms defined the position of the membrane bilayer; *ii)* the distribution of peptide atoms defined the position of the peptide; *iii)* the distribution of all Lys side-chain atoms defined the distance of Lys residues from the bilayer.

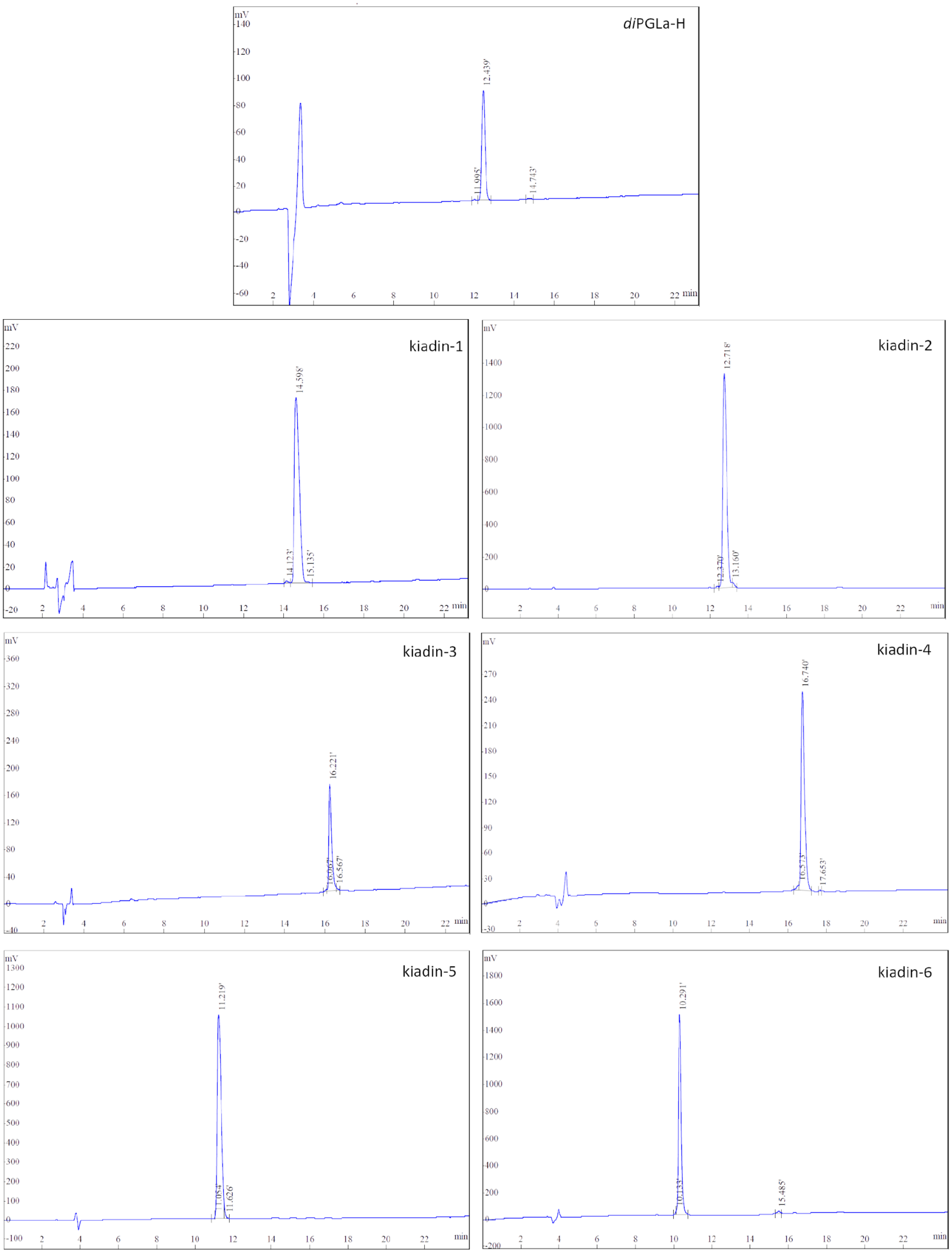

Figure S1A. Analytical RP-HPLC of kiadin peptides. Phenomenex Gemini-NX analytical column (C18, 5 µm, 110 Å, 4.6 x 250 mm) was used with 15-50 % acetonitrile/0.1% TFA gradient in 25 min with flow of 1.0 mL/min.

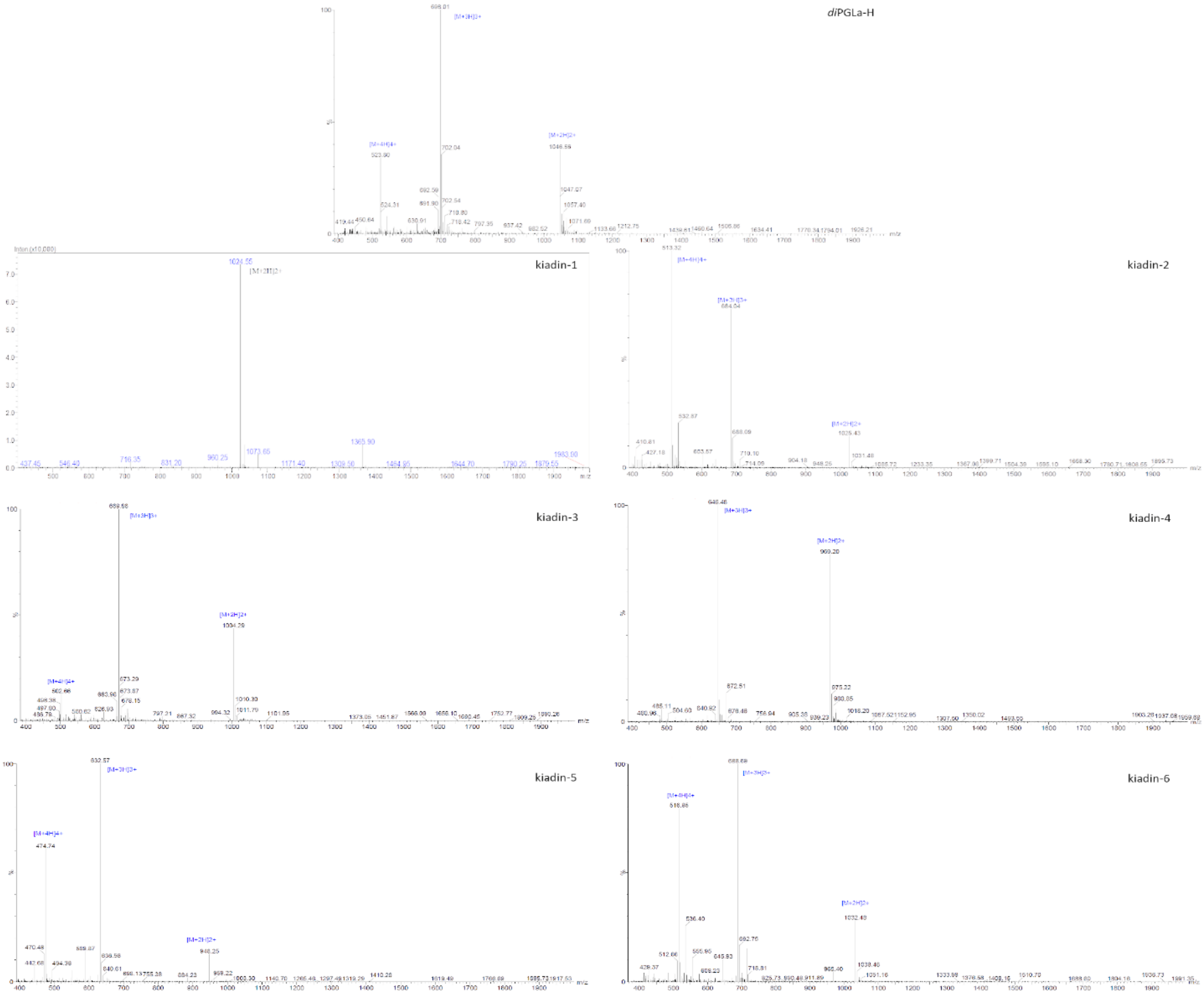

Figure S1B. ES-MS spectra for kiadins peptides. The m/z spectra were provided by Genic Bio who synthesized the peptides, on a Waters either a Waters ZQ2000 (*di*PGLa-H and kiadins-2 to -6) or Shimadzu LC-MS 2020 (kiadin-1). In both cases a flow of 0.2 ml/min was used, with a detector voltage 1.5 kV and a probe bias of +4.5 kV.

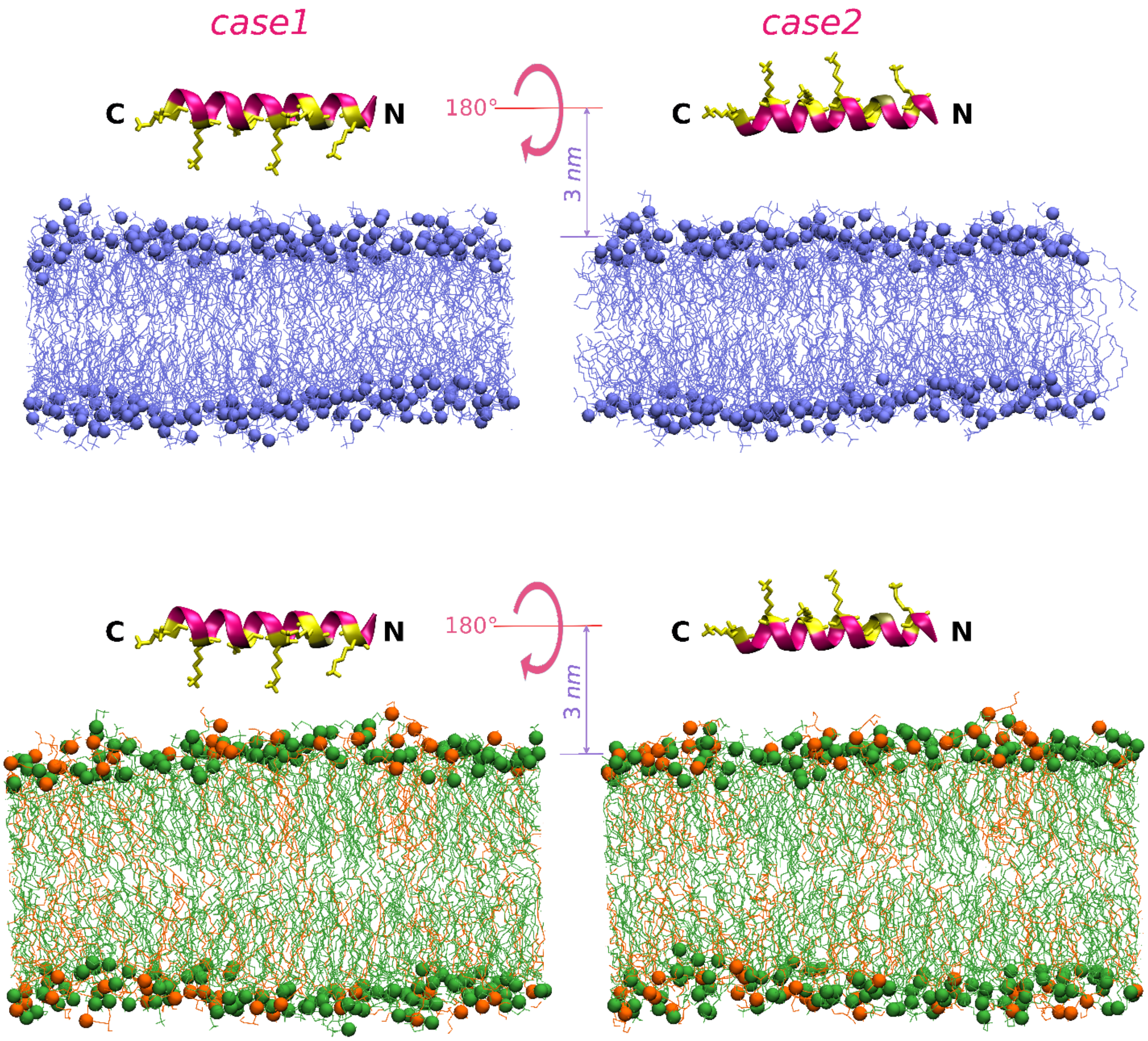

Figure S2. Snapshots of the initial conditions for MD simulations of kiadin peptides. Representation of the initial conditions for *case1* and *case2* simulation runs for the *di*PGLa-H peptide. The snapshots are taken at 0 ns simulation time (after the energy minimization procedure). The peptide structure is defined by the QUARK output [2,3]. In *case1*, the peptide was positioned parallel to and 2.5 nm distant from the surface of the lipids bilayer (distance between peptide center mass of average position of P atoms in upper leaflet) facing it with the polar surface. In *case2*, it was rotated 180° around the helical axis, to face the bilayer with its hydrophobic surface. Peptides are represented as ribbon models with the hydrophobic residues in magenta and polar ones in yellow, with Lys residue side chains shown as stick representations. Membrane phospholipids are shown in blue for DLPC membranes (top figures) and in green and orange respectively for POPE and POPG (lower figures), with phosphate groups represented as spheres and acyl chains as lines.

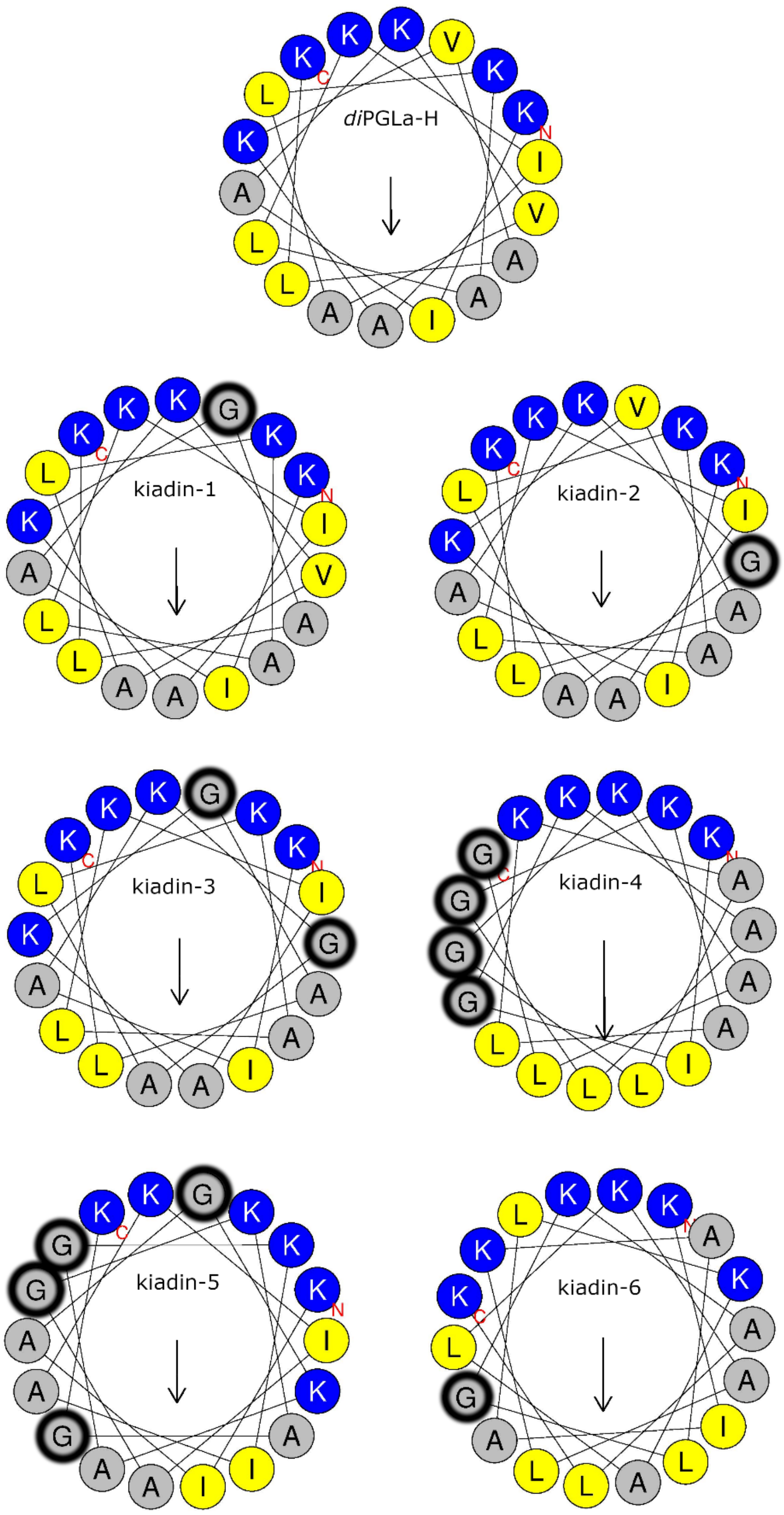

Figure S3. Helical wheel projections of kiadin peptides. Obtained using HeliQuest [4].

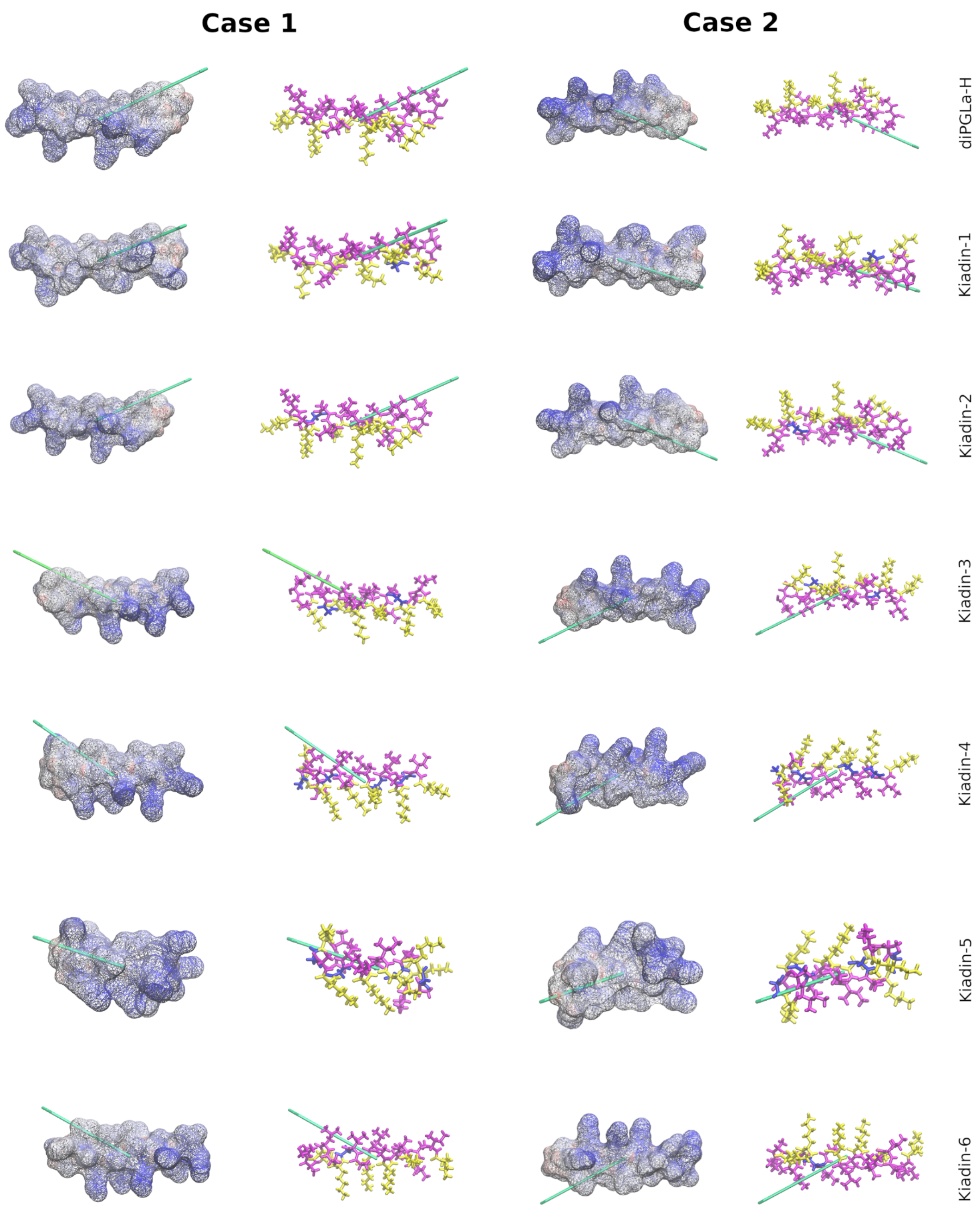

Figure S4. Visual representation of the 3DHM results for kiadins at 0 ns simulation time [5]. In this figure we show the representative structure of peptide as the electrostatic potential on the solvent-accessible surface and as the snapshot where polar residues are shown in yellow and hydrophobic in magenta color, of the same structure with its calculated 3D-HM vector (light green arrow). The left-hand side represents *case1* where peptide is facing the membrane with its polar side and the right-hand side *case2* when the hydrophobic side of peptide is towards the membrane, while each row represents the indicated peptide (on the right). Note that the structures for the two cases are subtly different after the equilibration step. The surface potential is represented using a transparent mode to make the 3D-HM vector visible.

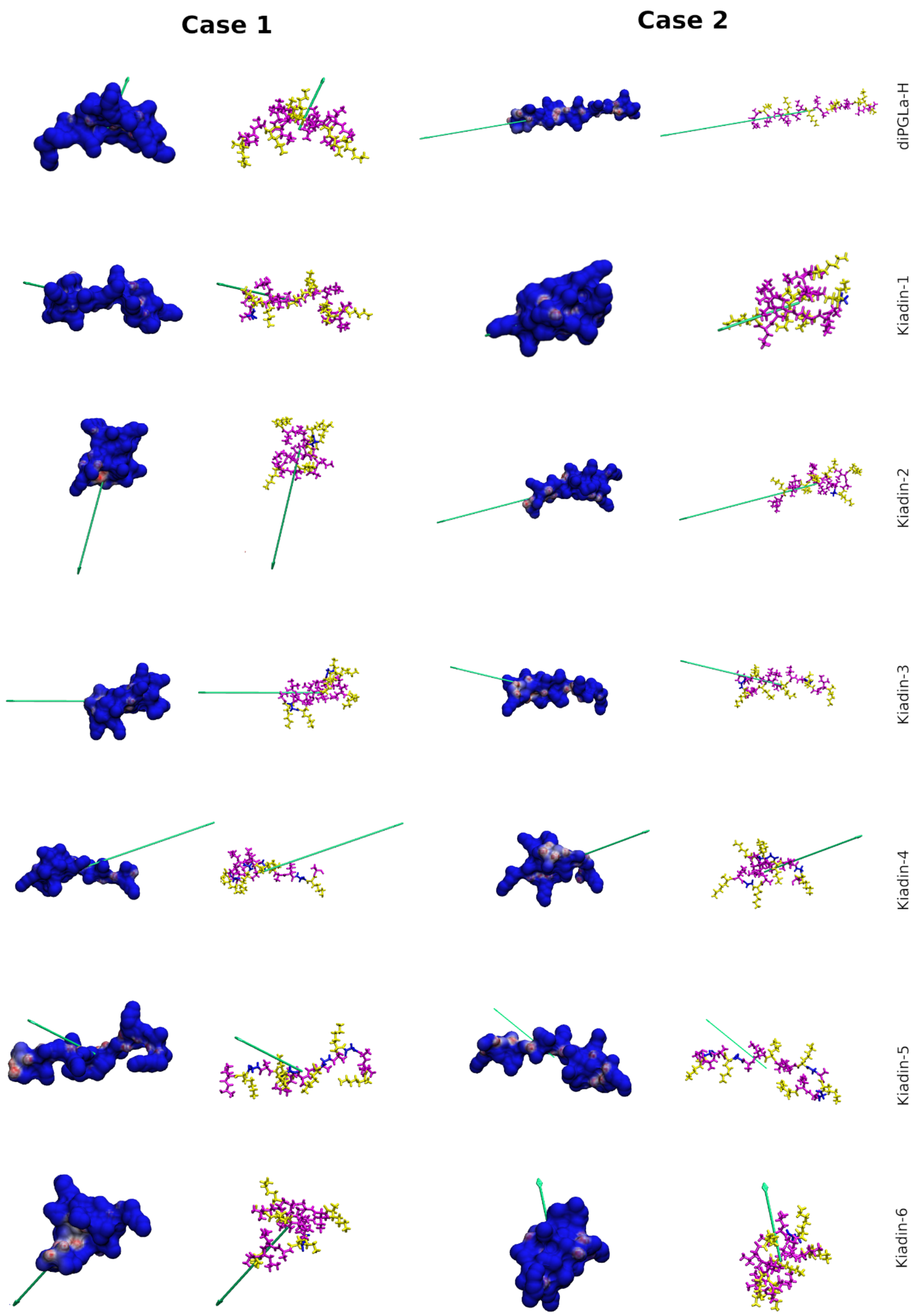

Figure S5A. The representation of the 3DHM results at 300 ns simulation time [5]. In this figure we show the representative structure of peptide interaction with DLPC membrane as the electrostatic potential on the solvent-accessible surface and as the snapshot where polar residues are shown in yellow and hydrophobic in magenta color, of the same structure with its calculated 3D-HM vector (light green arrow). The left-hand side represents *case1* where peptide is facing the membrane with its polar side and the right-hand side *case2* when the hydrophobic side of peptide is towards the membrane, while each row represents the indicated peptide (on the right).

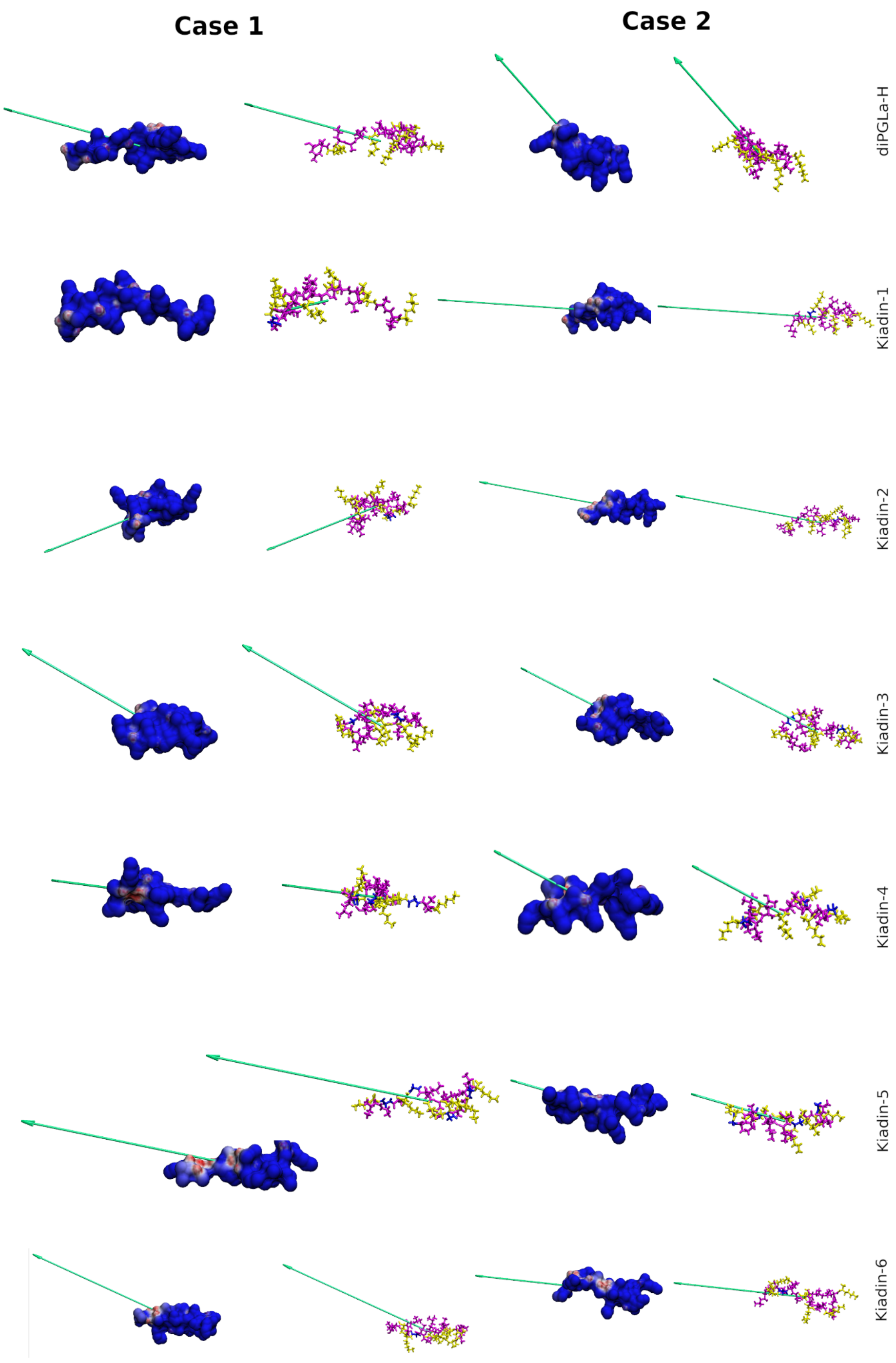

Figure S5B. The representation of the 3DHM results at 300 ns simulation time [5]. In this figure we show the representative structure of peptide interaction with POPE:POPG membrane as the electrostatic potential on the solvent-accessible surface and as the snapshot where polar residues are shown in yellow and hydrophobic in magenta color, of the same structure with its calculated 3D-HM vector (light green arrow). The left-hand side represents *case1* where peptide is facing the membrane with its polar side and the right-hand side *case2* when the hydrophobic side of peptide is towards the membrane, while each row represents the indicated peptide (on the right).

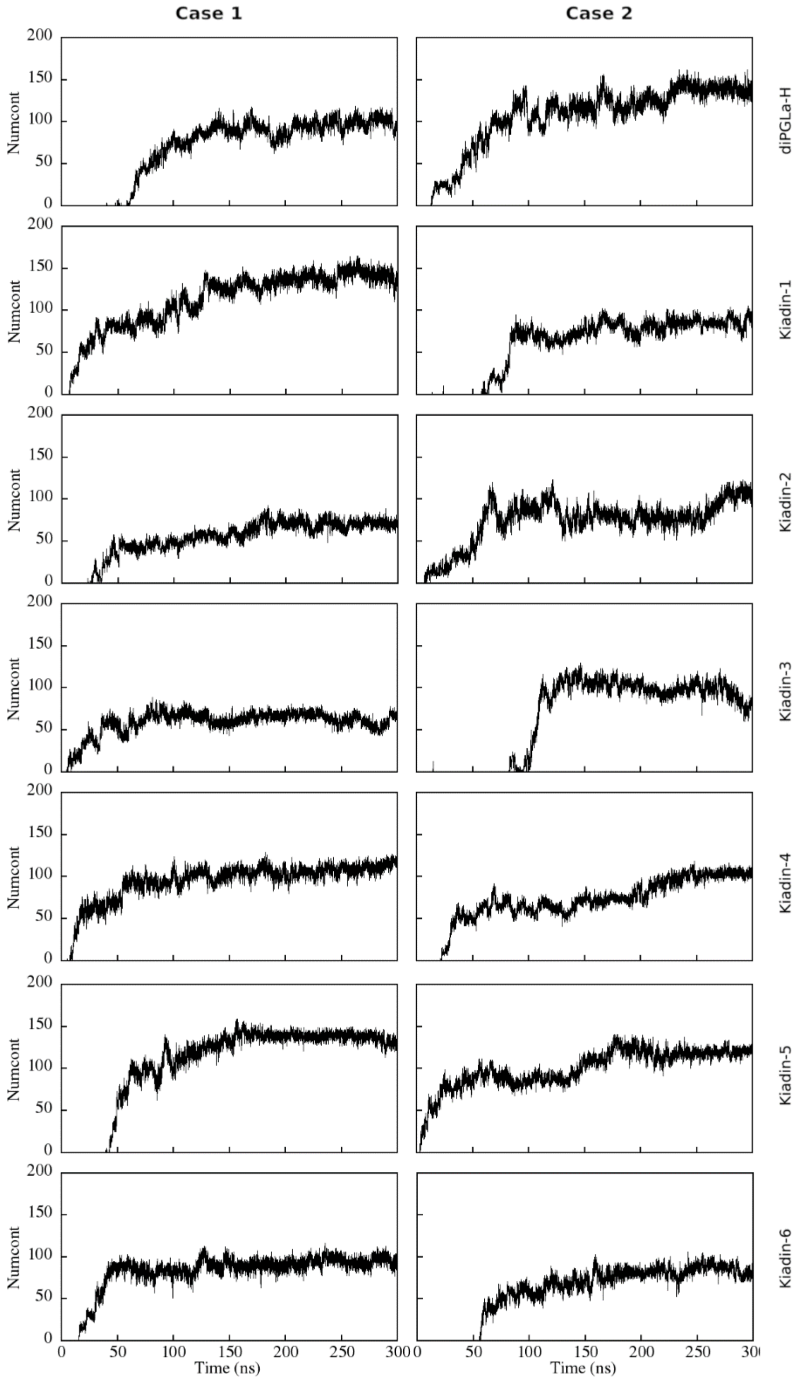

Figure S6A. Number of contacts as a function of simulation time between all peptide atoms and phospholipid phosphate atoms for DLPC systems. In this figure, we used the gmx routine mindist [1] to show the time dependence of the number of contacts between peptide and phosphate atoms of phospholipids, which were calculated using the cut-off distance of 0.6 nm. The multiple contacts between peptide' atoms and one phosphate atom are counted as one. From these plots one can derive the binding time for each peptide. The first column represents *case1*, and the second *case2* simulation runs, while each row shows the results for each peptide (the names of the peptides are on the right side of the graphs).

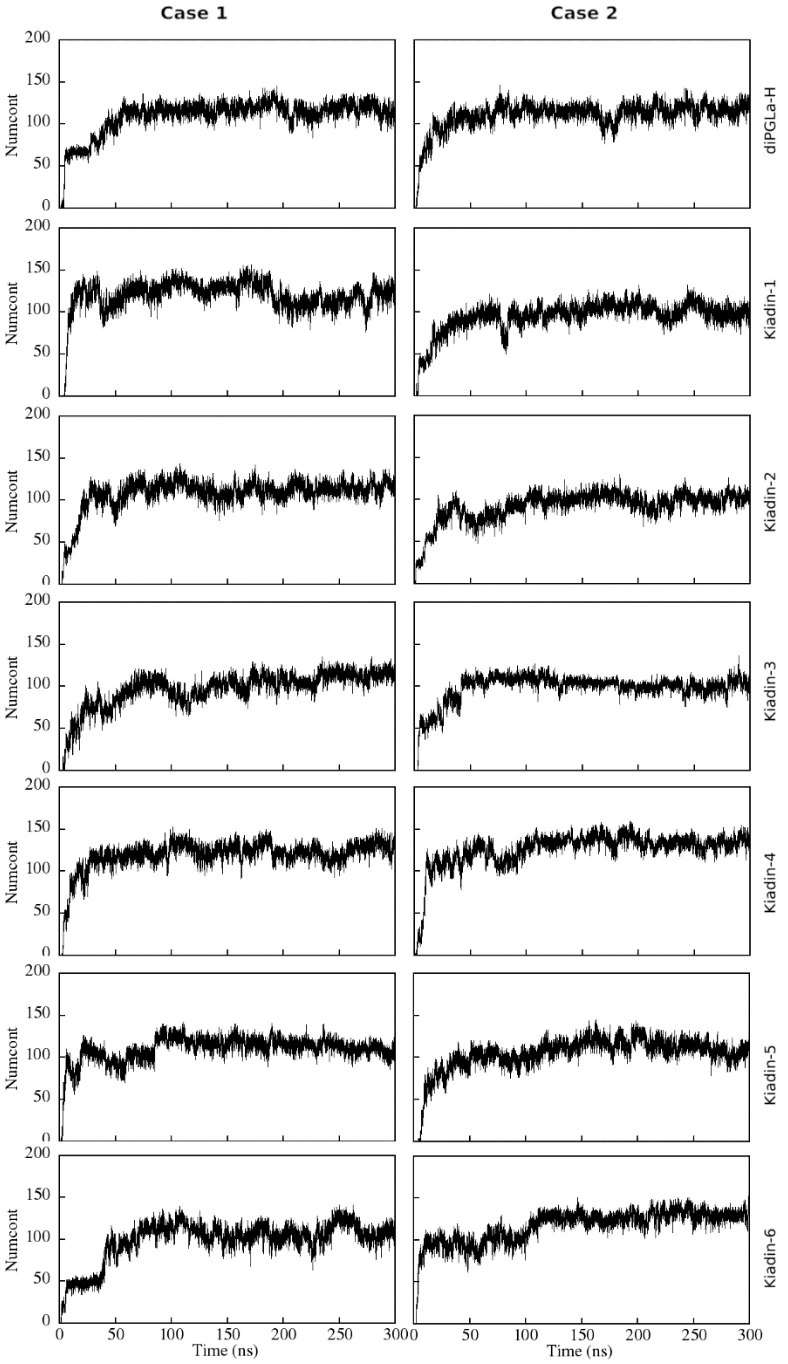

Figure S6B. Number of contacts as a function of simulation time between all peptide atoms and phospholipid phosphate atoms for POPE:POPG systems. In this figure, we used the gmx routine mindist [1] to show the time dependence of the number of contacts between peptide and phosphate atoms of phospholipids, which were calculated using the cut-off distance of 0.6 nm. The multiple contacts between peptide' atoms and one phosphate atom are counted as one. From these plots one can derive the binding time for each peptide. The first column represents *case1*, and the second *case2* simulation runs, while each row shows the results for each peptide (the names of the peptides are on the right side of the graphs).

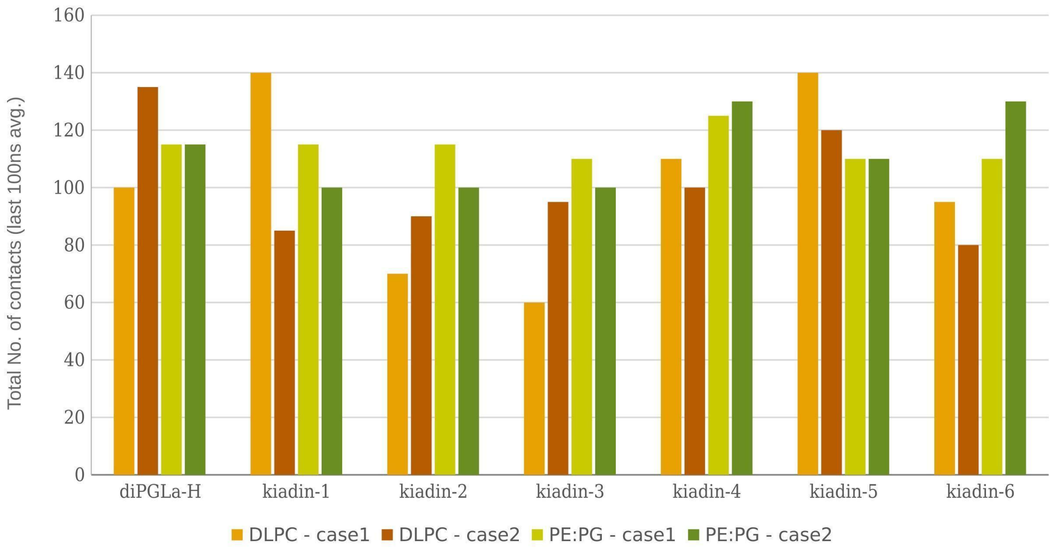

Figure S7. Average number of contacts between all atoms belonging to the peptide and phosphate atoms of phospholipids averaged over the last 100 ns of simulation time [1]. Results were derived from those shown in Figures S6A and S6B. For each peptide, histograms show the average number of contacts for *case1* and *case2* and each type of membrane, as indicated. The names of the peptides are shown under each set of histograms.

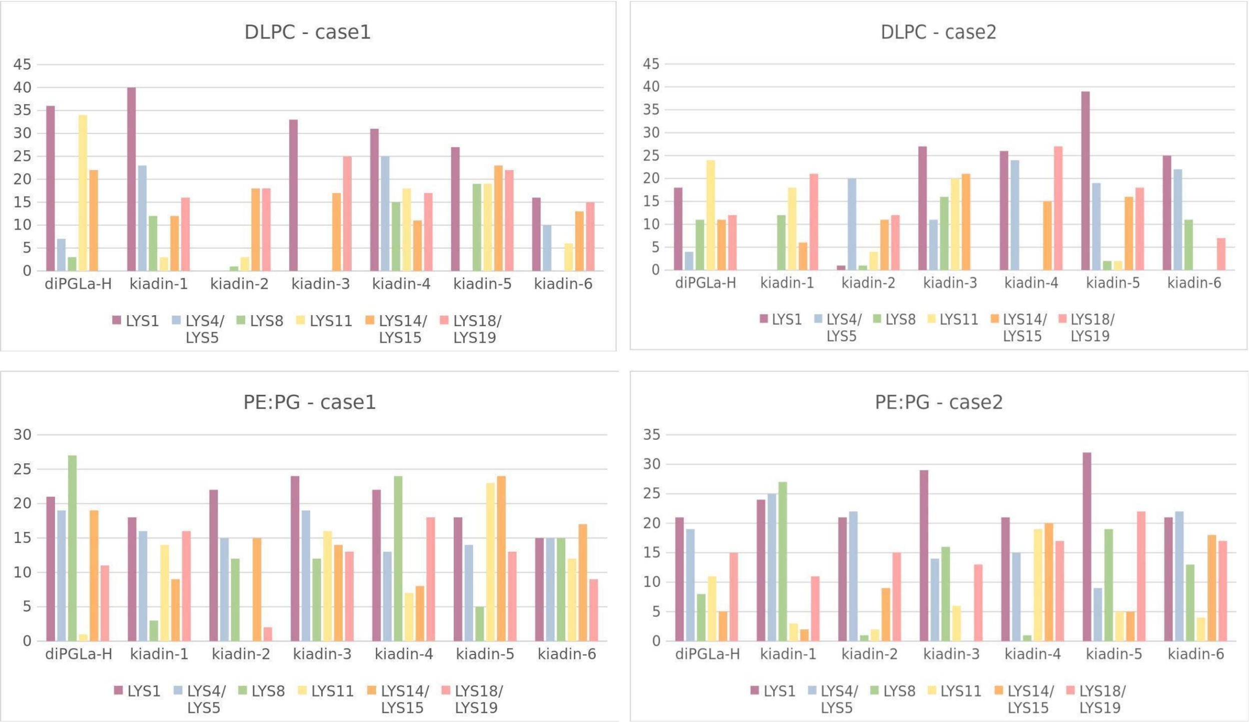

Figure S8. Average number of contacts between individual peptide Lys side-chains and membrane phosphate atoms averaged over the last 100 ns of simulation time. The contacts were calculated with the gmx routine mindist [1] with the cut-off distance of 0.6 nm, and each contact between any Lys side-chains' atoms and any phosphate atom are counted as one. For each peptide, histograms show the average number of contacts for *case1* and *case2* and each type of membrane, as indicated. The color code for each Lys side-chain is shown under the histograms.

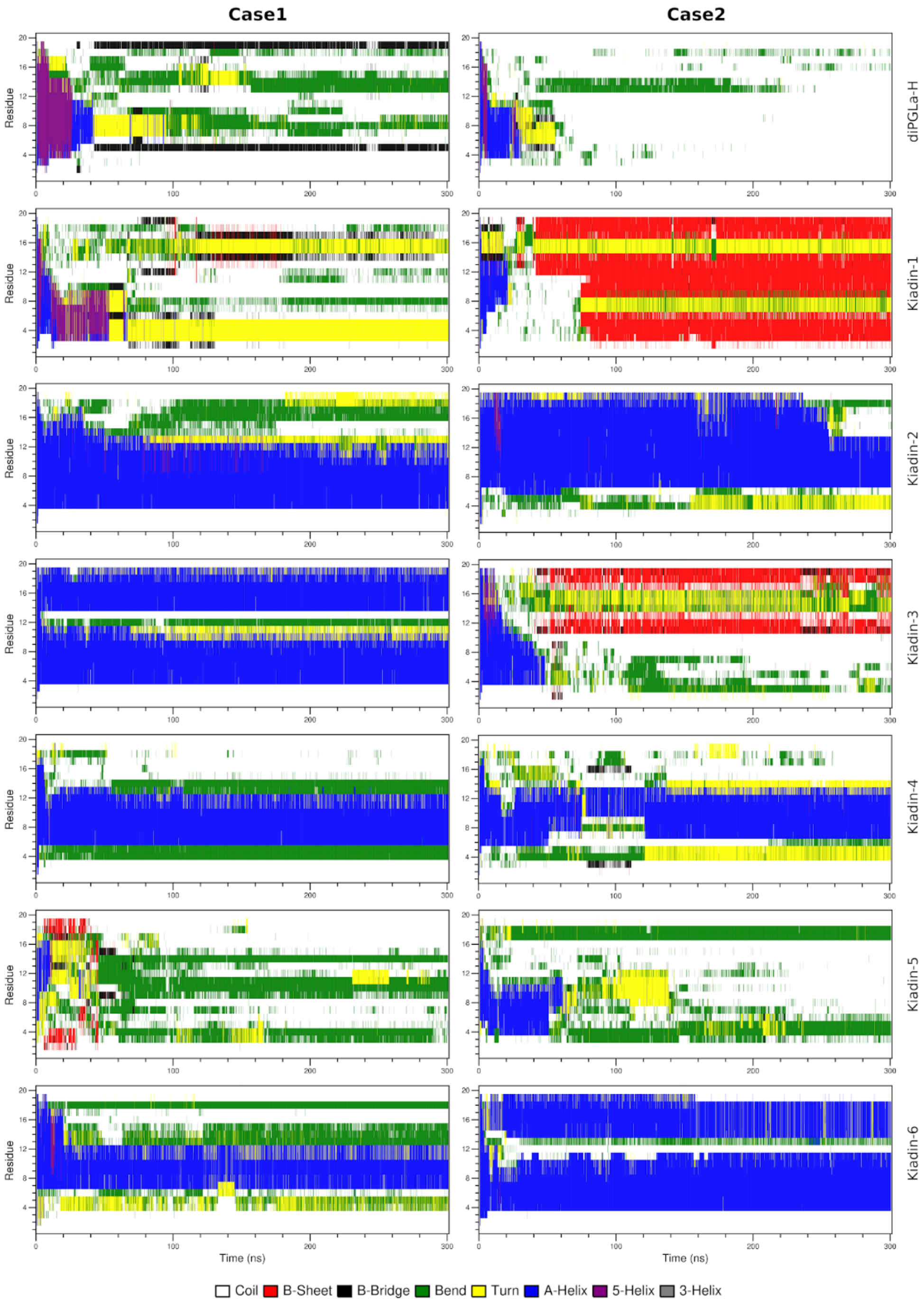

Figure S9A. The time evolution of the secondary structuring for 300 ns in DLPC systems. The plots are calculated by the DSSP program [6]. The first column shows the simulation data for *case1* experiment and the second column for *case2* runs. Each row represents data for each peptide (the peptide's names are on the outmost right). The DSSP assigns types of secondary structuring for each residue in the peptide and each time step, colored according to the legend included at the bottom of the figure. The starting structures were α-helical (see Figure S2) defined by the QUARK program, while the snapshot of the last conformations is shown in Figure S5A.

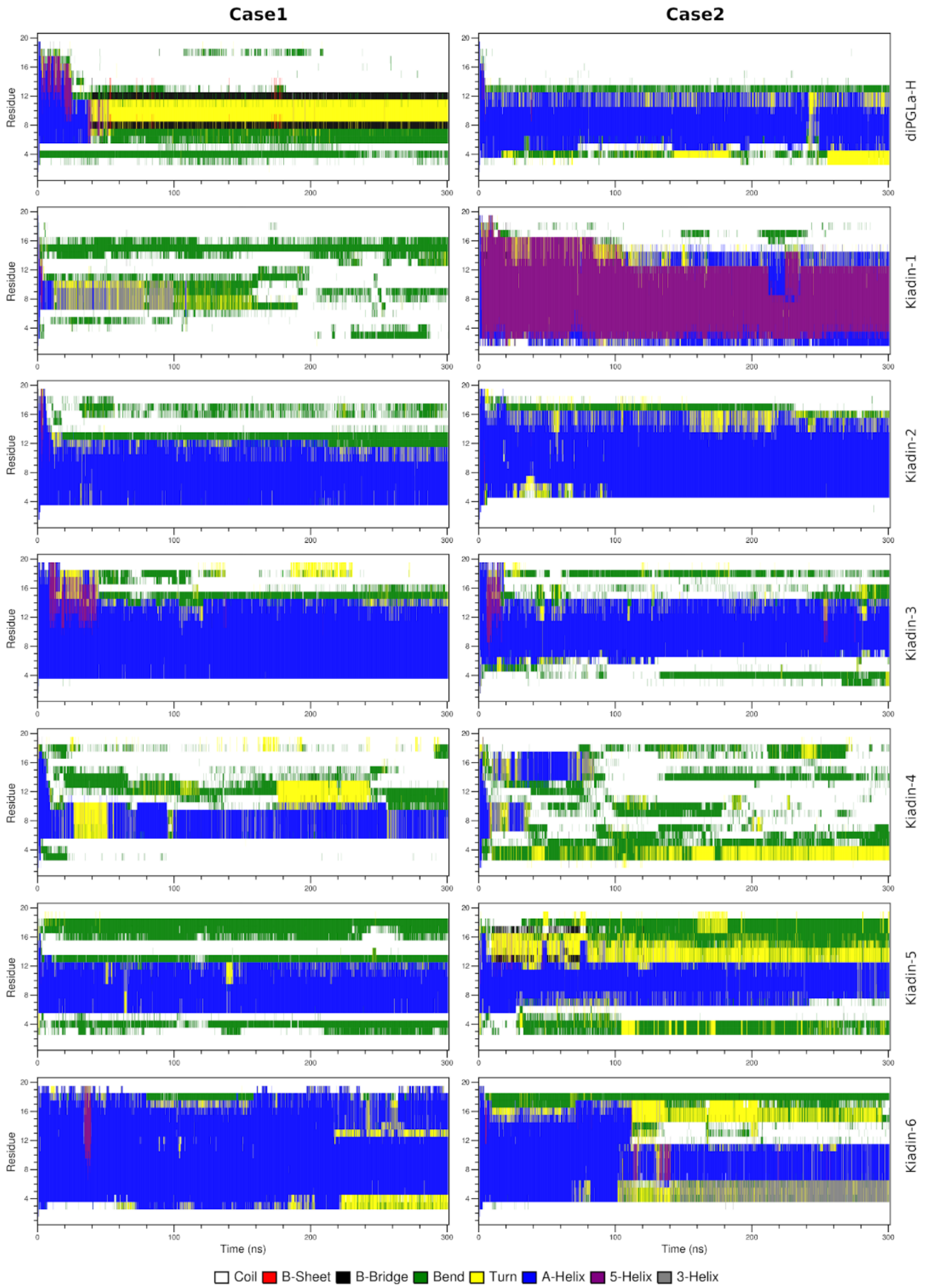

Figure S9B. The time evolution of the secondary structuring for 300 ns in POPE:POPG systems. The plots are calculated by the DSSP program [6]. The first column shows the simulation data for *case1* experiment and the second column for *case2* runs. Each row represents data for each peptide (the peptide's names are on the outmost right). The DSSP assigns types of secondary structuring for each residue in the peptide and each time step, colored according to the legend included at the bottom of the figure. The starting structures were α-helical (see Figure S2) defined by the QUARK program, while the snapshot of the last conformations is shown in Figure S5B.

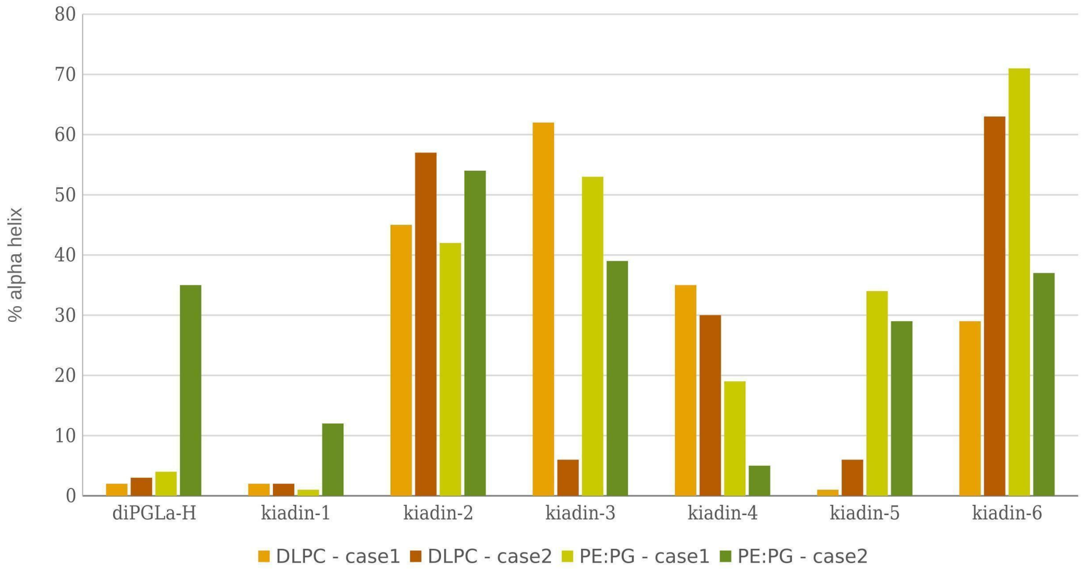

Figure S10. Percent α-helicity for each kiadin averaged over 300 ns simulation time. The % α-helicity was calculated using DSSP program [6] and is shown tabular and also as a histogram. The data for peptides interaction with DLPC membrane are shown in blue, while for peptides interacting with POPE:POPG membrane are in green colors, where *case1* (polar side initially facing membrane surface) are dark blue and light grey bars; *case2* (hydrophobic side initially facing membrane surface) are light blue and dark green bars.

Table S1. 3D-HM calculations [5]**.** Strength of 3D-HM and angle between 3D-HM vector and +z axis (axis perpendicular to the membrane surface), calculated at 0 ns and 300 ns simulation time.

	Module of 3DHM vector *[ÅkT/e]*					Angle between 3DHM vector and +*z* axis *[°]*					
		case1	*case2*	*case1*	*case2*	*case1*	*case2*	*case1*	*case2*	*case1*	*case2*
	H_2O	PE:PG		DLPC		H_2O		PE:PG		DLPC	
**Time** *[ns]*	**0**	**300**				**0**		**300**			
**Diel. const.**	**78.54**	**20**				**78.54**		**20**			
***di*PGLa**	**23.5 ± 0.5**	42.7	41.1	13.7	68.7	64.9 ± 0.1	115.3 ± 1.0	73.3	49.8	29.8	100.4
**kiadin-1**	**18.1 ± 0.7**	12.4	69.5	27.4	18.1	70.5 ± 1.1	108.8 ± 1.2	103.2	84.7	79.7	107.2
**kiadin-2**	**23.2 ± 0.9**	46.2	67.1	37.0	52.7	67.3 ± 0.9	113.7 ± 0.7	110.1	73.1	166.7	104.0
**kiadin-3**	**23.9 ± 0.9**	44.6	39.8	38.2	37.9	63.5 ± 0.8	115.6 ± 0.2	58.2	66.6	89.0	76.4
**kiadin-4**	**20.2 ± 0.4**	27.8	25.5	47.8	34.9	56.1 ± 0.6	122.5 ± 1.5	82.1	55.6	64.9	72.6
**kiadin-5**	**16.3 ± 0.8**	64.3	27.1	20.7	22.0	73.0 ± 1.1	107.9 ± 0.9	81.1	73.5	62.6	49.6
**kiadin-6**	**20.2 ± 0.7**	65.2	49.9	26.0	20.3	60.8 ± 0.1	119.9 ± 1.7	67.8	85.4	134.7	25.6

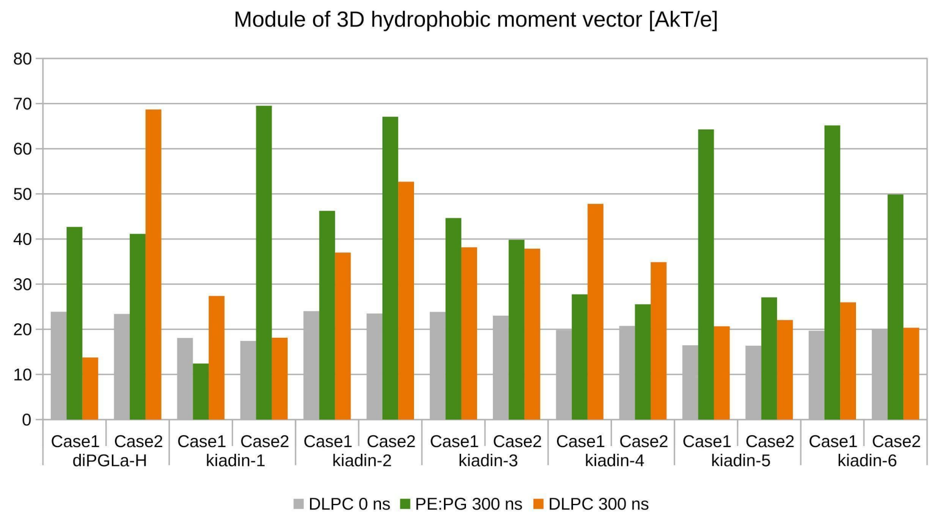

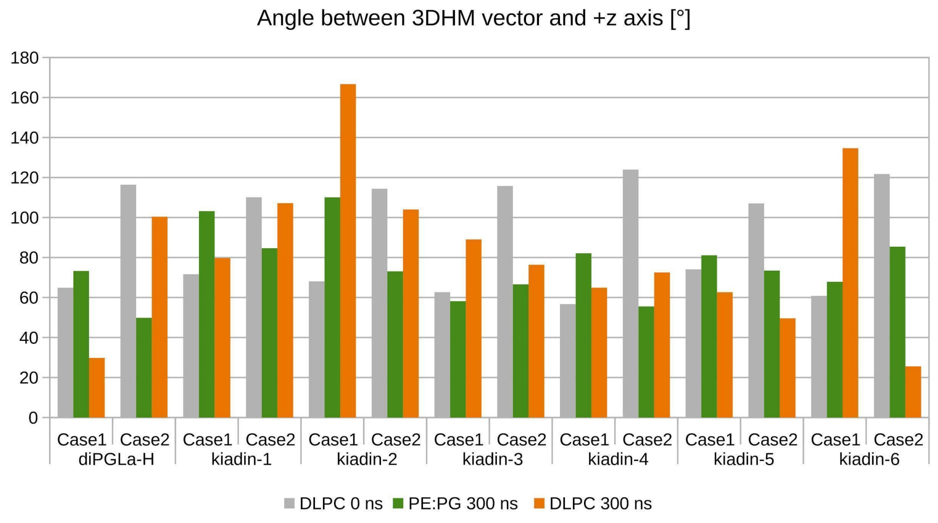

Figure S11. 3D-HM properties: top graph strength of 3D-HM and bottom graph angle between 3D-HM vector and +z axis (axis perpendicular to the membrane surface), calculated at 0 ns and 300 ns simulation time [5].

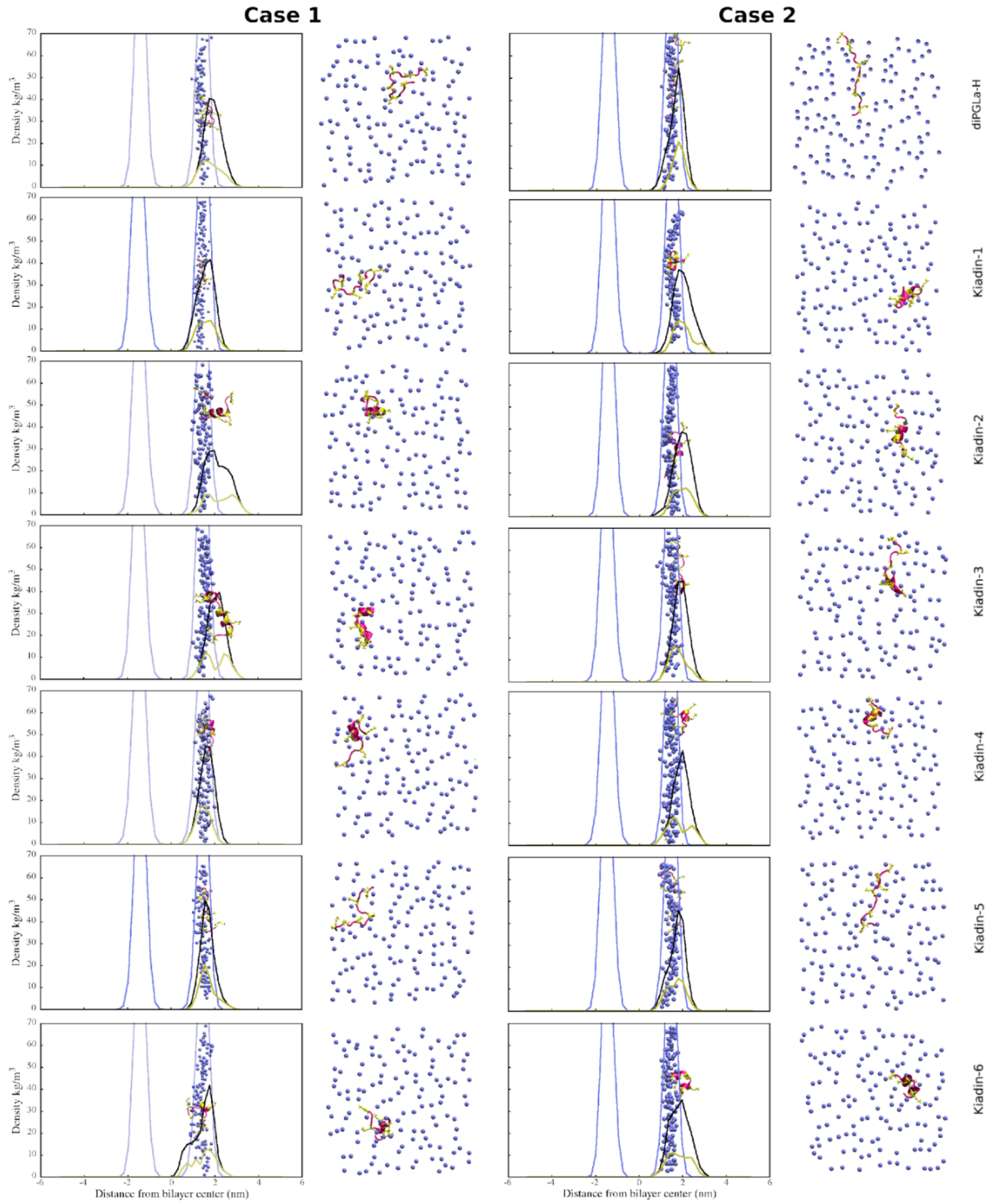

Figure S12A. Density profiles for kiadin peptides with DLPC membrane sampled over the last 100 ns simulation time and calculated by *gmx density* [1]. The black curve shows the mass distribution of all atoms in peptides relative to the bilayer, represented by the phosphorus atom distribution (blue curves). The yellow curves instead show the distance of Lys residues/side-chains atoms from the bilayer. The structure snapshots are conformations of the peptide at the membrane surface at 300 ns (end of simulation), where blue balls represent Ps from DLPC lipids, with the side (in the profiles) and top view. The left-hand side represents *case1* and the right-hand side *case2*, while each row represents the indicated peptide (on the right).

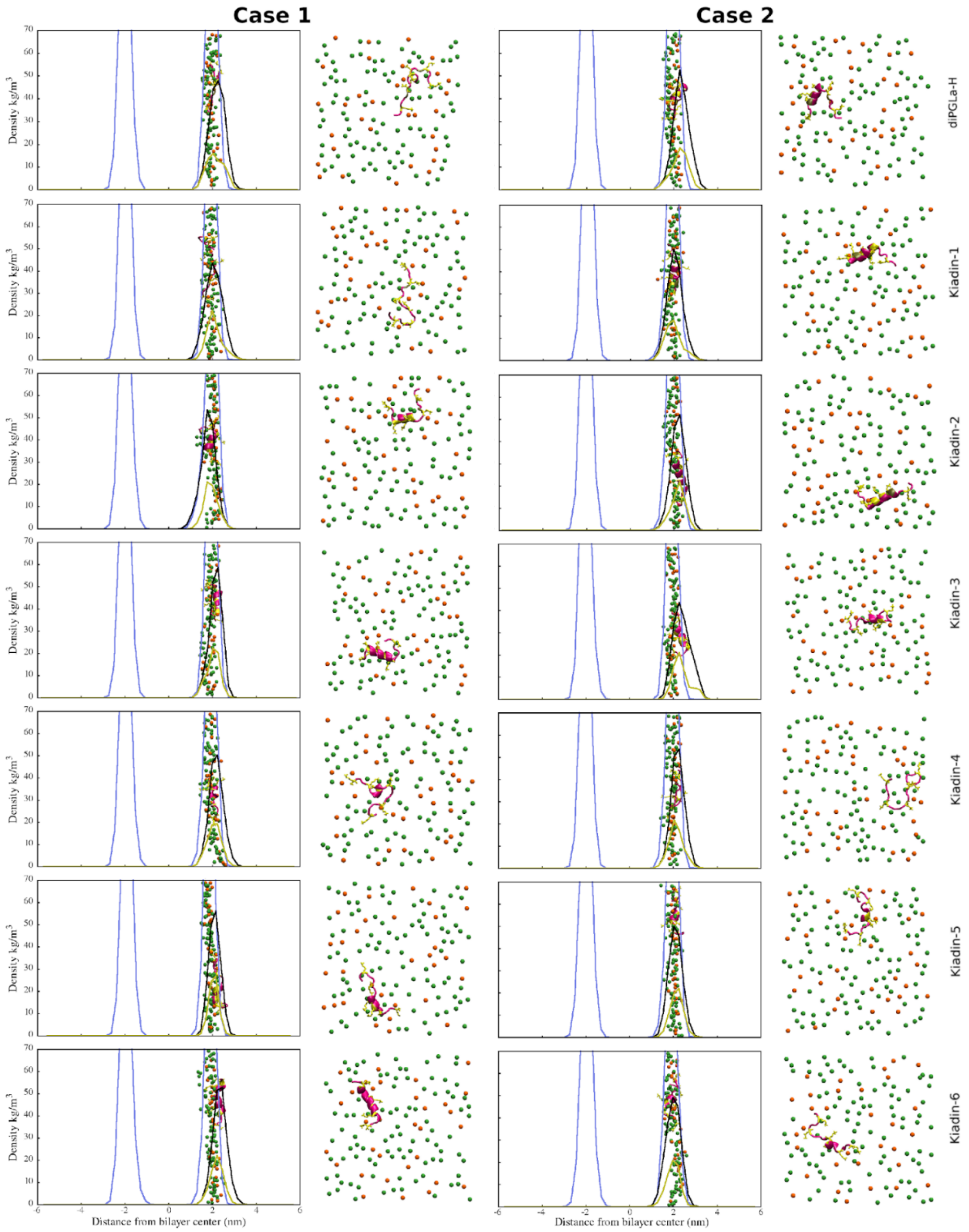

Figure S12B. Density profiles for kiadin peptides with POPE:POPG membrane sampled over the last 100 ns simulation time and calculated by *gmx density* [1]. The black curve shows the mass distribution of all atoms in peptides relative to the bilayer, represented by the phosphorus atom distribution (blue curves). The yellow curves instead show the distance of Lys residues/side-chains atoms from the bilayer. The structure snapshots are conformations of the peptide at the membrane surface at 300 ns (end of simulation), where green balls represent Ps from POPE, and orange Ps from POPG lipids, with the side (in the profiles) and top view. The left-hand side represents *case1* and the right-hand side *case2*, while each row represents the indicated peptide (on the right).

Table S2. Qualitative comparison of MD simulation, biophysical data and biological activities. Note that ++++ corresponds to the strongest effect, and – no effect. RBC, high hemolytic effect; MEC-1, high damage to cells; Antimicrobial potency, low MIC value; Structuring, large conformational change; Binding, large change in RU; MD helicity, % calculated α-helicity; 3D-HM, size of hydrophobic moment vector; Contacts, N° of contacts between all peptide atoms and all membrane phosphate atoms; Insertion, overlap between peptide density profile and membrane phosphate density profile. For MD results, the contributions from both *case1* and *case2* are considered.

	Cytotoxicity		Antimicrobial potency		Structuring (CD)		Binding	MD helicity		3D HM		Contacts		Insertion	
	RBC	MEC-1	*E coli*	*S aureus*	(LUV-)	(LUV°)	(LUV°)	DLPC	PE/PG	DLPC	PE/PG	DLPC	PE/PG	DLPC	PE/PG
*di*PGLa-H	+++	++++	+++	++++	+	-	+++	-	+	+++	++	++++	+++	++	++
kiadin-1	+++	+++	++++	+++	+++	-	++++	-	-	+	+++	+++	+++	++++	+++
kiadin-2	++	++	++++	+	++++	-	++	+++	+++	+++	++++	+	+++	++	+++
kiadin-3	+	++	++	+	++++	-	++	++	+++	++	++	+	+++	++	++
kiadin-4	++++	+++	+	+	++	-	+++	+	+	++	+	+++	++++	+++	++
kiadin-5	-	nd	-	-	++++	-	+	-	++	+	+++	++++	+++	+++	++
kiadin-6	++++	++++	+++	++	++	++	++++	++++	++++	+	++++	++	++++	++++	+++

References

1. Abraham, M.J.; Murtola, T.; Schulz, R.; Páll, S.; Smith, J.C.; Hess, B.; Lindahl, E. GROMACS: High Performance Molecular Simulations through Multi-Level Parallelism from Laptops to Supercomputers. SoftwareX 2015, 1–2, 19–25, doi:10.1016/j.softx.2015.06.001.
2. Xu, D.; Zhang, Y. Toward Optimal Fragment Generations for Ab Initio Protein Structure Assembly. *Proteins* **2013**, *81*, 229–239, doi:10.1002/prot.24179.
3. Xu, D.; Zhang, Y. Ab Initio Protein Structure Assembly Using Continuous Structure Fragments and Optimized Knowledge-Based Force Field. Proteins: Structure, Function, and Bioinformatics 2012, 1715–1735, doi:10.1002/prot.24065.
4. Gautier, R.; Douguet, D.; Antonny, B.; Drin, G. HELIQUEST: A Web Server to Screen Sequences with Specific α-Helical Properties. Bioinformatics 2008, 24, 2101–2102, doi:10.1093/bioinformatics/btn392.
5. Reißer, S.; Strandberg, E.; Steinbrecher, T.; Ulrich, A.S. 3D Hydrophobic Moment Vectors as a Tool to Characterize the Surface Polarity of Amphiphilic Peptides. Biophys J 2014, 106, 2385–2394, doi:10.1016/j.bpj.2014.04.020.
6. Kabsch, W.; Sander, C. Dictionary of Protein Secondary Structure: Pattern Recognition of Hydrogen-Bonded and Geometrical Features. Biopolymers 1983, 22, 2577–2637, doi:https://doi.org/10.1002/bip.360221211.

Graphical tools: VMD (Humphrey, W.; Dalke, A.; Schulten, K. VMD: Visual Molecular Dynamics. Journal of Molecular Graphics 1996, 14, 33–38, doi:10.1016/0263-7855(96)00018-5), Microsoft Excel and Gnuplot.
